# Gelatin Improve Storage Stability of Alginate-Encapsuled Krill Oil Microcapsules

**DOI:** 10.3390/foods15040711

**Published:** 2026-02-14

**Authors:** Xuan Ma, Jiangying Heng, Xian Zhang, Zhihua Zhang, Hongshuai Sun, Yiming Wei, Yi He, Bo Liu, Yu Zhang, Jing Jin, Tao Wei, Zhuo Zhao, Wenjie Yan, Feng Wang

**Affiliations:** 1 College of Biochemical Engineering, Beijing Union University, Beijing 100101, China; maxuan@buu.edu.cn (X.M.); 15999227335@163.com (J.H.); m18997735052@163.com (Z.Z.);; 2 China Green Food Development Center, Ministry of Agriculture, Beijing 100101, China; 13369100612@163.com (X.Z.);; 3 Weifang Hairun Huachen Testing Technology Co., Ltd., Weifang 261061, China; 4 Beijing Key Laboratory of Bioactive Substances and Functional Food, Beijing Union University, Beijing 100101, China

**Keywords:** antarctic krill oil (AKO), encapsulation, sodium alginate (ALG), gelatin (GLN), storage stability

## Abstract

Antarctic krill oil (AKO) is a valuable nutraceutical; however, it is highly susceptible to oxidation. Encapsulation represents an effective strategy to enhance the storage stability of AKO. This study explored a novel approach for encapsulating AKO using sodium alginate (ALG) and gelatin (GLN) to improve its stability, and multiple parameters were systematically evaluated, including oil-loading efficiency, surface oil content, particle size, water activity, and thermal stability. Additionally, core-material retention efficiency, acid value, peroxide value, and anisidine value were measured after accelerated oxidation. The results demonstrated that the optimal encapsulation conditions consisted of an ALG:GLN ratio of 2:1, a 9% CaCl_2_ coagulation bath, 750 μm nozzle size, followed by freeze-drying. Under these conditions, the microcapsules achieved an oil-loading efficiency of 62.63% and a surface oil content of 19.21%. The water activity of the microcapsules was 0.516. Thermogravimetric analysis indicated that AKO microcapsules encapsulated with ALG/GLN exhibited higher thermal stability (~300 °C) compared to those encapsulated with ALG alone (~280 °C). When AKO or its microcapsules were subjected to accelerated oxidation at 65 °C, compared to ALG-encapsulation alone, the ALG/GLN encapsulation system significantly reduced the oxidation indicators of the oil, such as acid value (24%), peroxide value (26%), and anisidine value (28%). In conclusion, incorporating GLN into ALG-based microcapsules significantly enhanced the antioxidant capacity of AKO and prolonged its shelf life.

## 1. Introduction

Antarctic Antarctic krill oil (AKO) is one of the world’s largest biomass sources and is rich in long-chain (LC) ω-3 polyunsaturated fatty acids (PUFAs) such as eicosapentaenoic acid (EPA, 20:5 *n*−3) and docosahexaenoic acid (DHA, C22:6 *n*−3), as well as the antioxidant astaxanthin [1,2]. In the last decade, AKO was explored for nutraceutical. AKO was approved or authorized by United States Food and Drug Administration (FDA) in 2008, Euro-pean Union (EU) in 2009 and Chinese government in 2014, as a GRAS (Generally Recognized as Safe) or valuable food, and EU has approved the use of AKO in foods for pregnant and lactating women [3,4].

However, the bioactive components of AKO, particularly polyunsaturated fatty acids (PUFAs) and astaxanthin, are susceptible to degradation by oxygen, light, heat and metal ions, resulting in deterioration of oil quality and off-flavors [5]. Oxidation of DHA and EPA produces hydroperoxides and unpleasant odors to consumers, and oxidation of astaxanthin causes discoloration [6,7]. AKO is susceptible to lipid oxidation during processing and storage, leading to nutrient loss and the formation of off-flavors and toxic compounds [8,9].

The development of various encapsulation systems for AKO to improve nutritional value, antioxidant capacity, and bioavailability has become a research hotspot. For example, C.A. Ortiz Sánchez et al. encapsulated AKO using gum arabic, achieving an optimal encapsulation efficiency of 47.6715% with an astaxanthin content of 38.5726 μg/g [10]. In addition, our laboratory previously explored krill oil Pickering emulsions prepared by whey protein isolate and chitosan, which achieved an encapsulation efficiency of 80.43% and high ferric reducing antioxidant power (a 2.8-fold increase) [11].

Among the polymers used as a membrane in the capsules, sodium alginate (ALG) is suitable in oil encapsulation because of its high gelling capacity, biocompatibility and low toxicity [12]. ALG is an anionic polysaccharide, in the presence of calcium ions, the ALG macromolecules crosslink to form a three-dimensional net-work called hydrogel to encapsule oil well [12,13]. However, the feasibility and storage stability of AKO encapsulation using ALG remain undetermined.

Gelatin (GLN), has been widely employed as a wall material for encapsulating various oils, such as ω-3 fatty acids [14,15]. Despite its advantages, its low intensity and high brittleness make it rarely used alone; instead, it is often used after modification through several methods, such as cross-linking [16,17]. GLN demonstrates strong attractive interactions with ALG, leading to the formation of interfacial complexes [18,19]. This interaction inhibits droplet coalescence and gravitational separation, significantly enhancing the stability of microemulsions. Previous studies have validated the efficacy of ALG and GLN in encapsulating oils, effectively enhancing their storage stability [20]. However, it’s important to note that different oils can induce substantial variations in the emulsification process [21].

In this study, we first determined the optimal process for encapsulating AKO using GLN and ALG. Subsequently, we systematically compared GLN/ALG-encapsulated and ALG-encapsulated AKO microcapsules, focusing on key properties such as particle size and water activity. To evaluate the storage performance of the microcapsules, we simulated product storage under accelerated oxidation conditions. By monitoring parameters including core-material retention efficiency, acid value, peroxide value, and anisidine value, we estimated the effective shelf-life of the microcapsules. Additionally, thermogravimetric analysis was conducted to assess the protective efficacy of the microcapsules under extreme temperatures. Overall, this study aimed to determine whether the addition of GLN could enhance the storage stability of ALG-encapsulated AKO microcapsules.

## 2. Materials and Methods

### 2.1. Materials

Antarctic krill oil (AKO) was provided by Aker Bio Marine Co., Lysaker, Norway. ALG (200 bloom) and GLN (180 bloom) were purchased from Macklin, Shanghai, China. Tween 80, calcium chloride (CaCl_2_) and petroleum ether were also obtained from Macklin Co., Ltd., Shanghai, China. Hydrochloric acid (HCl) was purchased from Tianjin Damao Chemical Reagent Factory, Tianjin, China.

### 2.2. Microcapsule Preparation

**Figure 1:**
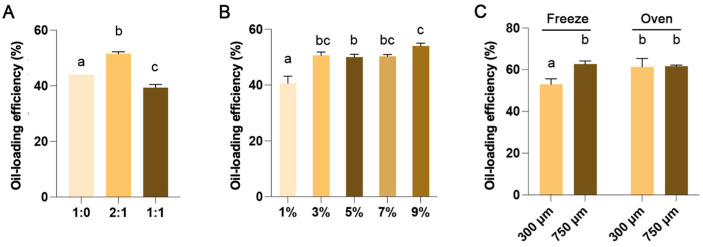
Effect of encapsulation process on oil-loading rate of ALG/GLN microcapsule. Effect of different (**A**) ALG/GLN ratio, (**B**) CaCl2 concentration, (**C**) vibrating nozzle diameter and drying method on oil-loading rate of ALG/GLN microcapsule. Data are presented as mean ± SD (n = 3). Different letters indicate significant differences between groups (*p <* 0.05).

The initial method for encapsulating AKO was previously described in Figure 1 of Reference [22]. Briefly, GLN was dissolved in distilled water and heated in a microwave oven. Subsequently, 3% (*m*/*v*) ALG was dispersed into the GLN solution, and the mixture was stirred and homogenized at 7000 r/min for 3 min using a shear homogenizer (#T25, IKA, Staufen, Germany) to obtain a homogeneous dispersion of ALG and GLN. Tween 80 was then added to the mixture to a final concentration of 1% (*m*/*v*), followed by thorough mixing. Next, 6% (*m*/*v*) krill oil was incorporated into the mixture, which was then homogenized again. Thereafter, the homogenized emulsion was subjected to high-pressure homogenization (#AH-NANO, ATS Nanotechnology Co., Ltd., Suzhou, China) at 500 bar and 4 °C for three cycles to obtain a well-homogenized, stable emulsion.

Microencapsulation was performed using a microencapsulator (#B390, BUCHI, Essen, Germany) with a flow rate of 10 mL/min, a frequency of 800 Hz, a voltage of 1 500 kV, and a stirring speed of 100 rpm. The resulting solution was injected into the coagulation bath by a syringe needle with the diameter size of 1.6 mm to form microencapsulation. The coagulation bath was composed of 5% (*w*/*v*) CaCl_2_ aqueous solution. After coagulation for 30 min, the wet gel beads were collected by filtration. Wash 3 times with distilled water to remove excess CaCl_2_. Then these gel beads were washed with distilled water and dried for 72 h in a freeze dryer (#FD-304, Jinan Junde Co., Ltd., Jinan, China).

### 2.3. Single-Factor Experiment

Based on the microcapsule preparation method described in Section 2.2, single-factor experiments were conducted to investigate the effects of three key variables on the microencapsulation efficiency, with oil-loading efficiency and surface oil content as the evaluation indicators. The detailed experiment was as following:

(1) The ratio of ALG to GLN (*m*/*m*) was set as the first single factor, with three levels: 1:0, 2:1, and 1:1. Other preparation conditions remained consistent with those in Section 2.2. (2) On the basis of the optimal ALG:GLN ratio (2:1) obtained from the above experiment, the concentration of CaCl_2_ aqueous solution was selected as the second single factor, with the CaCl_2_ concentration set at 1%, 3%, 5%, 7%, and 9% (*w*/*v*). All other experimental parameters were strictly consistent with those in Section 2.2. (3) The combination of nozzle size and drying method was chosen as the third single factor. Two nozzle diameters (300 μm and 750 μm) and two drying methods (oven drying and freeze drying) were selected, forming four experimental groups: 300 μm nozzle + oven drying, 300 μm nozzle + freeze drying, 750 μm nozzle + oven drying, and 750 μm nozzle + freeze drying. For all groups, the ALG:GLN ratio was fixed at 2:1 and the CaCl_2_ concentration was fixed at 9%. Other preparation parameters remained unchanged.

The particle size, water activity, thermal stability, and oxidative stability of AKO-encapsulated microcapsules were determined using the microcapsules (#B390, BUCHI, Essen, Germany) prepared under the following conditions determined by Single-factor experiment: ALG:GLN ratio of 2:1, 9% CaCl_2_ coagulation bath, 750 μm nozzle size, and subsequent freeze-drying.

### 2.4. Surface Krill Oil of Microcapsules

The content of free (surface) AKO (w_f_) was determined according to the method reported by Liu Shi [23]. 3 g AKO microcapsule sample was placed in a screw-capped flask. Then, 27 mL of petroleum ether was added to extract the surface AKO. The extraction was carried out at ambient temperature using an incubator shaker (#THZ-D, Suzhou Peiying Experimental Equipment Co., Suzhou, China) at 100 r/min for 10 min. After extraction, the dispersion was filtered. The filtrate containing the surface AKO was collected and evaporated to dryness in a fume hood at ambient temperature. w_f_ was determined gravimetrically. The filtrate containing the microcapsule was collected, freeze dried, and then weighed (w_m_).

### 2.5. Oil-Loading Efficiency of Microcapsules

Oil-loading efficiency = w_t_/w_m_. The total krill oil (w_t_) included both w_f_ and microencapsulated AKO. First, 1 g sample was mixed with 15 mL HCl (4 mol/L) in an incubator shaker at 200 r/min for 30 min to disintegrate AKO microcapsules. Then, 10 mL of petroleum ether was added and the mixture was shaken at 150 r/min for 12 h to extract the krill oil. The dispersion was filtered, and the filtrate was collected and evaporated in a fume hood. All these procedures were performed at ambient temperature. The w_t_ content was determined gravimetrically.

### 2.6. Particle Size

The size of the microcapsules was evaluated using a microscope (#CX43, Olympus China Co., Ltd., Beijing, China) (n = 10). An optical microscope was used to capture images for observing the appearance, and then the ImageJ software (version 1.54f, NIH, Bethesda, MD, USA) was employed to measure the particle sizes of the microcapsules.

### 2.7. Water Activity

The diffusion method specified in the national food safety standard GB 5009.238-2016 [24] “Determination of Water Activity in Food” was adopted, and a water activity meter (#HD-5, Wuxi Huake Instrument Co., Ltd., Wuxi, China) was used to determine the water activity of the microcapsules.

### 2.8. Thermogravimetric Analysis

Under a nitrogen flow rate of 30 mL/min, 5 mg sample was placed in the thermogravimetric analyzer, and the temperature was increased from 30 to 600 °C at a heating rate of 10 °C/min. The mass change, mass loss rate of the sample, as well as the endothermic and exothermic changes of the sample were detected by a thermogravimetric analysis instrument (#Pyris Diamond TG/DTA, Pyris Diamond, Perkin Elmer Inc., Shelton, CT, USA), forming the Thermal Gravity Analysis (TG), Derivative of Thermal Gravity (DTG), and Differential Thermal Analysis (DTA) curves respectively.

### 2.9. Accelerated Oxidation Experiment

An oven was used to accelerate the oxidation of AKO or its microcapsules to evaluate their oxidative stability. Storing AKO or its microcapsules in an oven at 65 °C for one day was equivalent to storing them at room temperature for one month. 1.5 g microcapsules were placed in a sealed dark bottle and kept in the oven for 6 days. Samples were taken on days 0, 1, 3, and 6 to measure the core-material retention efficiency, acid value, peroxide value, and anisidine value.

The acid value was determined using the first method (cold solvent indicator titration method) described in the national food safety standard “GB 5009.229—2016 [25] Determination of acid value in food”. The peroxide value was measured using the titration method described in the national food safety standard “GB 5009.227—2016 [26] Determination of peroxide value in food”. The anisidine value was measured according to the standard “GB/T 24304—2009 [27] Determination of anisidine value in animal and vegetable fats and oils”. The visible spectrophotometer (#V-1800) was purchased from Shanghai MAPADA Co., Ltd., Shanghai, China).

The core-material retention efficiency was calculated using the formula: Core material retention efficiency (%) = (Microencapsulation Core-material retention efficiency after heating/Microencapsulation Core-material retention efficiency before heating) * 100%. Microencapsulation efficiency refers to (w_t_-w_f_)/w_t_ × 100.

### 2.10. Statistical Analysis

Statistical analysis was performed using SPSS 2019 Statistics and drew using GraphPad Prism 8 software (GraphPad Software, San Diego, CA, USA). Descriptive parameters were expressed as mean ± SD. The Kolmogorov–Smirnov test was used to test whether data were normally distributed and whether the logarithmic transformation of non-normally distributed data rendered them normally distributed. Differences among normally distributed values of three experimental groups were analyzed by one-way ANOVA, followed by a Tukey multiple comparisons posttest. Differences between normally distributed values of two experimental groups were analyzed by an unpaired Stu dent’s *t*-test. Data involving multiple variables and time points were analyzed by two-way ANOVA. *p <* 0.05 was considered significant.

## 3. Results

### 3.1. Effect of Encapsulation Process on Oil-Loading Efficiency of ALG/GLN Microcapsule

Encapsulation process exerts a significant influence on the oil-loading efficiency, the potential mechanism inferred as follows: GLN dissolution and thermal unfolding in aqueous phase, the hydrophilic groups (e.g., -NH_3_^+^, -COO^−^, -OH) on GLN chains orient outward and interact with water molecules, the hydrophobic side chains (including hydrophobic amino acid residues and aliphatic segments) tend to aggregate inward to avoid contact with water, forming nanoscale hydrophobic domains (or micelle-like aggregates) in the aqueous solution. After ALG addition, local electrostatic complexation occurs between the positively charged regions (e.g., -NH_3_^+^) of GLN and the -COO^−^ groups of ALG, thereby forming a polyelectrolyte complex. In this composite system, ALG serves as a highly hydrophilic backbone, while gelatin contributes excellent amphiphilicity, emulsifying capacity, and flexibility, which collectively improve the interfacial adsorption performance between the wall material and oil phase. After adding tween 80 (which contains approximately 20 hydrophilic polyoxyethylene units and one hydrophobic fatty acid tail), its hydrophilic terminal groups form hydrogen bonds with the -OH and -COO^−^ groups of ALG. When AKO added, the hydrophobic terminal groups of Tween 80 anchor firmly to the oil phase. After adding CaCl_2_, Ca^2+^ migrate from the aqueous phase to the surface of emulsion droplets and coordinate with the free -COO^−^ groups on ALG chains, which leading to the gradual crosslinking of ALG molecules. Eventually, a microcapsule membrane with a highly crosslinked surface is formed, which can effectively encapsulate AKO within the microcapsule core. The nozzle diameter size effects the initial droplet size and shape during microcapsule formation, and drying method effects the structural integrity and oil migration behavior during the final solidification stage.

To optimize the oil-loading efficiency of this microcapsule, it is necessary to systematically determine the key processing parameters based on the above-described formation mechanism.

Krill oil is excellent nutritious, however, it has an unpleased taste. Therefore, it is more suitable for encapsulation in large-sized microcapsules, which show better leak tightness [28]. Large-sized microcapsules are often associated with a high oil-loading efficiency [29]. Hence, the oil-loading efficiency, which refers to the ratio of the amount of capsule’s AKO to the weight of the whole microcapsule, was used as the first indicator to screen the optimal preparation process for AKO microcapsules. A higher oil-loading efficiency indicates a better encapsulation effect and a more efficient utilization of the microcapsule wall material to retain the krill oil. As wall materials, ALG endows microcapsules with excellent mechanical strength, while GLN imparts elasticity and compactness, enabling microcapsules to be easily shaped into various forms. When ALG and GLN are combined in an appropriate ratio, they can act synergistically [30]. Different ALG:GLN ratio were tested, results indicated that the highest oil-loading efficiency (51.45%) was achieved with a ALG to GLN ratio of 2:1 (Figure 1A). This may be attributed to the higher tensile strength and breaking elongation at this ratio. As reported in the literature, the best values of the tensile strength and breaking elongation of blend fibers were obtained when gelatin content was 30 wt % of ALG [18].

ALG and GLN can undergo ionic cross-linking in the presence of Ca^2+^. We hypothesis the Ca^2+^ concentration has significant effect on the oil-loading efficiency of microcapsule. Hence, we explored the impact of CaCl_2_ concentration on the oil-loading efficiency under the conditions where the ratio of ALG to GLN was fixed at 2:1. As depicted in Figure 1B, when the CaCl_2_ concentration was increased from 1% to 9%, the oil-loading efficiency increased from 40.50% to 54.13%. Based on these results, a 9% CaCl2 solution was chosen for subsequent experiments.

Tween 80 is a typical nonionic surfactant widely used in food emulsification and microencapsulation [31]. Numerous studies have confirmed that tween 80 exhibits superior emulsifying performance in lipid encapsulation systems using ALG or GLN as wall materials, compared with CTAB, SDS, and Span 80 [32]. Therefore, tween 80 was chosen as the emulsifier in this study. The commonly used concentration range of tween 80 in ALG or GLN-based microencapsulation is 1–2%. Previous reports demonstrated that 1% tween 80 significantly reduced the interfacial tension (from 37.86 mN/m to 19.76 mN/m) and decreased droplet size in gelatin-stabilized emulsions, and this effect was not strongly dependent on tween 80 concentration [33]. For ALG-based microcapsules, tween 80 improved creaming stability of ALG dispersions but did not significantly change particle size or creaming index (CI) of emulsions [31]. Given the non-concentration dependence of tween 80 within the effective range and its well-documented optimal performance at 1%, tween 80 concentration was not included as a variable in the single-factor experiments. Consider the strongly effect of tween 80 on droplet size and interfacial stabilization, investigation of tween 80 concentration should be considered in future studies.

To investigate the impact of nozzle diameter sizes on the oil-loading efficiency of microcapsule, microcapsules were produced using an encapsulator equipped (#B390, BUCHI, Essen, Germany) with different size of vibrating nozzle diameter. Surprisingly, although a 300 μm nozzle produced smaller microcapsules, it resulted in significantly lower oil-loading efficiency compared to the 750 μm nozzle (62.63%) (Figure 1C—freeze method), implying the smaller diameter nozzle may create microcapsules with excessively large surface areas and low oil-loading efficiency.

To investigate the drying method on oil-loading efficiency of microcapsules, microcapsules were dried by freeze or oven, the results were shown in Figure 1C. Drying in an oven had no impact on the oil-loading efficiency of the microcapsules prepared using a 750-μm or a 300-μm vibrating nozzle (~61.30%). However, freeze-drying led to a decrease in the oil-loading efficiency of the microcapsules produced with a 300-μm nozzle. This suggests that the structural integrity of small-particle microcapsules might be relatively fragile, making them prone to damage by the ice crystals formed during the freeze-drying process.

### 3.2. Effect of Microencapsulation Process on Surface Oil Content of Microcapsule

**Figure 2:**
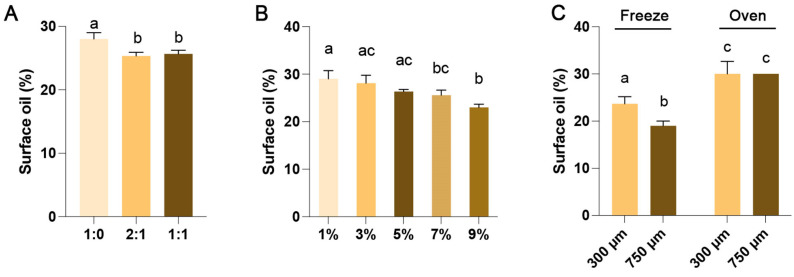
Effect of microencapsulation process on surface oil content of microcapsule. Effect of different (**A**) ALG/GLN ratio, (**B**) CaCl_2_ concentration, (**C**) vibrating nozzle diameter and drying method on surface oil content of microcapsule. Data are presented as mean ± SD (n = 3). Different lowercase letters above bars indicate significant differences (*p <* 0.05).

The surface oil refers to the AKO outside the microcapsule wall material. Excessive surface oil makes it easier for the microcapsules to adhere to each other, affecting the stability of the microcapsules; or it can accelerate the oxidation and deterioration of the AKO. Here, we examined the effects of the variables in 3.1 on the surface oil of the microcapsules, and the results are shown in Figure 2. When the ratio of ALG:GLN is 1:0, the content of surface oil is relatively high (27.95%), while the ratio of ALG:GLN is 2:1 or 1:1, the content of surface oil decreases (~25.70%) (Figure 2A).

It has been reported in the literature that CaCl_2_ migrates from the aqueous phase to the surface of microcapsules and cross-links with ALG, forming a membrane with a highly cross-linked surface and a relatively low cross-linking degree in the interior, thereby better encapsulating the core material [12]. The content of surface oil decreases as the concentration of CaCl_2_ increases (Figure 2B). Combined with the data of increased oil-loading efficiency, it is speculated that higher Ca^2+^ content leads to increased cross-linking and particle formation, which is consistent with previous reports in the literature [34]. The surface oil content reached 23.00% under the condition of 9% CaCl_2_. With the increase of calcium ion concentration, its cross-linking efficiency decreased significantly [34]. It was reported that ALG has a limited binding capacity for Ca^2+^, with approximately one Ca^2+^ binding to every eight carboxyl groups (or eight sugar units) of alginate. Through calculation, the molar ratio of –COOH (from ALG) to Ca^2+^ (from CaCl_2_) is approximately 1:5.4 when using 3% (*m*/*v*) ALG and 9% (*m*/*v*) CaCl_2_, indicating that 9% CaCl_2_ is sufficient for cross-linking. Therefore, no higher concentration of CaCl_2_ was set in this study.

The surface oil content of the microcapsules prepared with 300 μm and 750 μm vibrating nozzles is approximately 30% after oven drying, with no significant difference between the two groups. In contrast, freeze-drying significantly reduces the surface oil content of the microcapsules, especially those prepared using the 750 μm vibrating nozzle (19.21%) (Figure 2C). It is interesting that freeze-drying could reduce the surface oil content but did not affect the oil-loading rate of the microcapsules produced with a 750-μm nozzle. It is hypothesized that freeze-drying might cause AKO to transform from the β crystal form to the β’ crystal form. This transformation could result in a lower melting point of AKO, making it more fluid [35]. However, this speculation requires further verification through additional experiments.

Tween 80, a non-ionic surfactant, helps disperse AKO evenly by reducing interfacial tension during emulsification. It may interact with the ALG/GLN wall materials through hydrogen bonding, enhancing the flexibility and compactness of the encapsulation layer during dehydration [36]. Additionally, Tween 80 can act as a protective agent during freeze-drying, preventing micro-fissures in the wall matrix caused by ice crystal growth and reducing the migration of krill oil to the surface, thereby lowering surface oil content [37].

Taking into account the results of the oil-loading efficiency and surface oil content, the optimal parameters for the microencapsulation process were determined as follows: a ratio of ALG to GLN of 2:1, a 9% CaCl_2_ solution, a 750-μm vibrating nozzle, and freeze-drying.

### 3.3. GLN Has No Effect on the Particle Size and Water Activity of ALG-Capsuled AKO Microcapsules

**Figure 3:**
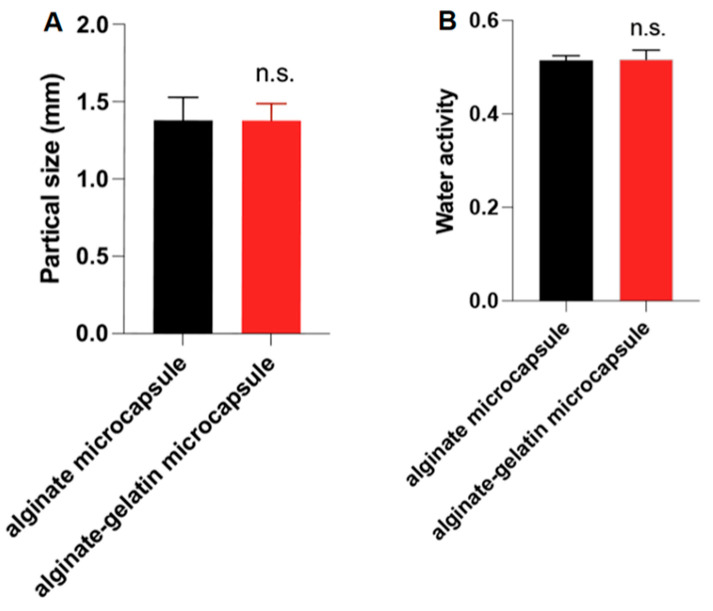
GLN has no effect on the particle size and water activity of ALG-capsuled AKO microcapsules. (**A**) The particle size (n = 10) and (**B**) the water activity (n = 3) of ALG-capsuled AKO microcapsules and ALG/GLN-capsuled AKO microcapsules. Data are presented as mean ± SD. n.s. indicates no significant difference between the groups (*p >* 0.05).

The particle size refers to the average diameter of the microcapsules. For nutritional supplements like AKO, which has an ordinary taste but is rich in nutritional value, microcapsules with large particle sizes can efficiently encapsulate AKO inside. This enables a slow release of the oil in the digestive tract of the body, prolonging the duration of the action of the nutritional components within the body. Here, we detected the particle sizes of the microcapsules encapsulated by ALG alone or by the combination of ALG and GLN. The results are shown in Figure 3A. Through the same preparation procedure, whether using ALG alone or in combination with GLN, the microcapsules of AKO can achieve the same particle size (~1.38 mm), indicating GLN cannot improve the particle size of ALG-capsuled AKO microcapsules.

Tween 80 was also added as a surfactant in this preparation process. It has been reported that adding 1% Tween 80 to the gelatin system caused a sudden decrease in surface tension (from 37.86 mN/m to 19.76 mN/m), thereby reducing the particle size of the system [33]. However, we did not specifically investigate the effect of Tween 80 on particle size in this study, which is worthy of further investigation in the future.

Water activity refers to the availability of water. The higher the water activity value, the higher the content of free water in the food, and the more easily the water can be utilized by microorganisms. Conversely, lower water activity value reflects a closer bound of water to other components, and less water is available for microorganisms. A water activity value lower than 0.600 will inhibit all biological activities, including those of fungi and bacteria, and prevent the spoilage of AKO microcapsules during storage and transportation. The experimental results are shown in Figure 3B. There is no significant difference in the water activity of AKO microcapsules encapsulated by ALG alone or in combination with GLN, both of which are ~0.516. This result is inconsistent with the fact that GLN has a higher water-retention ability, which is because the introduction of Ca^2+^ decreased the hydrophilicity of the microcapsules [18].

### 3.4. GLN Improves the Thermal Stability of ALG-Capsuled AKO Microcapsules

**Figure 4:**
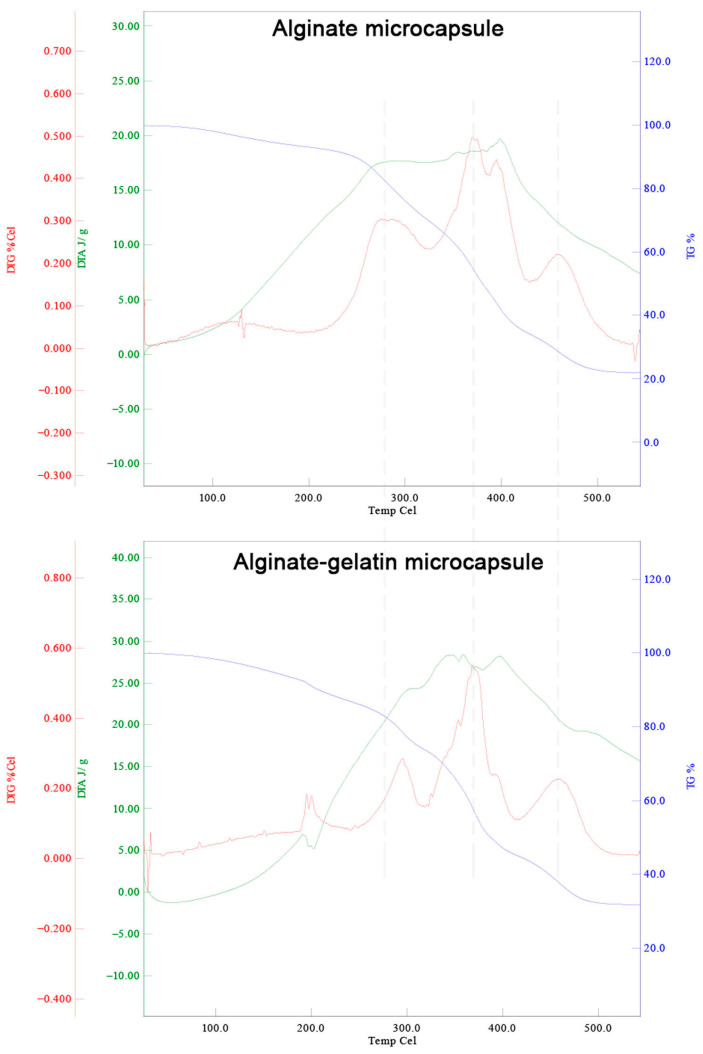
GLN improves the thermal stability of ALG-capsuled AKO microcapsules. The TG curve (blue), DTG curves (red), and DTA curves (green) of ALG capsuled AKO microcapsules (above) and ALG/GLN-capsuled AKO microcapsules (below).The vertical dashed lines are provided as visual guides to facilitate the alignment and comparison of thermal transition peaks between the two samples.

In industrial such as food and pharmaceuticals production, microcapsules might undergo high-temperature processing, including spray-drying, baking, and spray-drying. Thermogravimetric analysis serves as an effective way to simulate these processes. By observing mass changes at different temperatures, researchers can gain insights into the stability of microcapsules under high-temperature conditions and the volatilization of core materials. The sample temperature was increased in a gradient from 30 °C to 600 °C to obtain the TG, DTG, and DTA curves. When observing the TG curve, a significant point can be found, that is, the temperature point at which the weight of the sample decreases most significantly. This indicates that at this temperature, the decomposition reactions of the sample are most active. The DTG is derived from the TG analysis, which reflect the influence of temperature change on the rate of weight change. The DTA curve mainly focuses on the heat change of the sample during temperature change, which helps to understand the endothermic or exothermic process of the reaction. The changes in the above curves of the ALG or ALG/GLN capsuled microcapsules were determined, and the results are presented in Figure 4. The TG curve (blue) reveals that ALG/GLN microcapsules retained more mass compared to ALG-only microcapsules across the temperature range. The DTG curves (red) shows that ALG microcapsules started significant thermal decomposition at ~280 °C, in contrast, ALG/GLN microcapsules remained stable until ~300 °C. The DTA curves (green) shows that compared to ALG microcapsules, the exothermic peak of ALG/GLN microcapsules occurred at a higher temperature, indicating a delay in the decomposition-related exothermic process. These results indicate that the addition of GLN can shift the starting temperature of the greatest degradation of the microcapsules to higher temperatures, which can mitigate the structural damage to microcapsules and the loss of core materials caused by high temperatures. This is consistent with the relevant literature [18]. The enhanced thermal stability might be due to the crystalline domains and the strong intermolecular interactions between ALG and GLN [18].

### 3.5. GLN Improves the Oxidative Stability of ALG-Capsuled AKO Microcapsules

At the initial stage of oil storage, unsaturated fatty acids in the oil undergo oxidation reactions due to contact with oxygen in the air, which results in the formation of primary oxidation products such as hydroperoxides, causing a rapid increase in the peroxide value. As storage time extends, hydroperoxides further decompose to yield secondary oxidation products like aldehydes and ketones. The presence and quantity of these secondary products are reflected by the anisidine value. Additionally, the hydrolysis of oil generates free fatty acids, causing an increase in the acid value. This increase may be accompanied by the emergence of off-odors and harmful substances, thereby undermining the quality and safety of products. According to the standard SC/T3506-2020 [38], the acid value of krill oil should not exceed 15 mg/g. The Global Organization for EPA and DHA Omega-3 (GOED), an industry-standard-setting organization, recommends that the maximum limits for the peroxide value and anisidine value of krill oil be 2.5 mmol/kg and 20 respectively [39].

**Figure 5:**
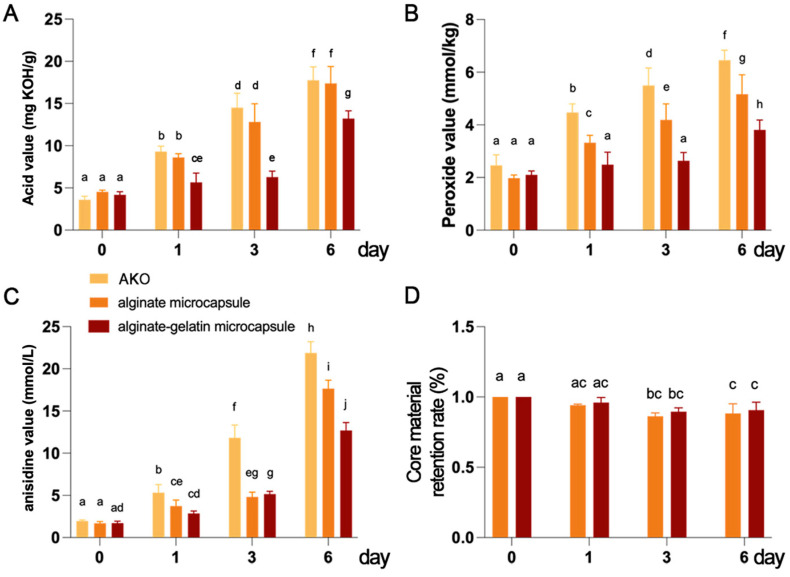
GLN improves the oxidative stability of ALG-capsuled AKO microcapsules. (**A**) Acid value, (**B**) peroxide value, and (**C**) anisidine value of krill oil, AKO microcapsules encapsulated by ALG, and AKO microcapsules encapsulated by ALG/GLN were determined during 6-day storage at 65 °C. (**D**) The core material retention rate of AKO microcapsules encapsulated by ALG or ALG/GLN were determined during 6-day storage at 65 °C. Data are presented as mean ± SD (n = 3). Different lowercase letters indicate significant differences between groups or time points (*p <* 0.05).

AKO or its microcapsules were placed in an oven at 65 °C for 0, 1, 3, and 6 days to simulate the storage conditions at room temperature for 0, 10, 30, and 60 days, respectively. The changes in acid value, peroxide value, and anisidine value were detected, and the results are shown in Figure 5A–C. On day 0, the baseline values of unencapsulated AKO, microcapsules with ALG as the single wall material, and ALG/GLN microcapsules were identical. As the storage time increased, the acid value, peroxide value, and anisidine value of unencapsulated AKO increased significantly. The peroxide value exceeded the standard limit on the 10th day (simulated by 1 day of oven storage), while the anisidine value and acid value exceeded the limits on the 60th day (simulated by 6 days of oven storage). Encapsulation with ALG alone as the wall material could effectively reduce the peroxide value and anisidine value of AKO, but had no significant effect on the acid value. This implies that the microcapsules have efficiency as an oxygen barrier, but their water barrier property may be general, which requires further verification through additional experiments. In contrast, encapsulation with the ALG/GLN composite wall material could significantly reduce the acid value, peroxide value, and anisidine value of AKO, and its protective effect was superior to that of ALG alone. This indicates that the addition of GLN enhanced the oxygen and water barrier capabilities of the microcapsules. Specifically, after 10 days of simulated storage (1 day of oven storage), ALG-only encapsulation could mitigate the increase in the peroxide value of AKO, yet the peroxide value still exceeded the standard. In contrast, ALG/GLN encapsulation could maintain the peroxide value within the standard range. After 30 days of simulated storage (3 days of oven storage), the peroxide values of AKO microcapsules encapsulated with ALG alone or ALG/GLN both exceeded the standard. Both ALG-only and ALG/GLN encapsulations could effectively prevent the anisidine value of AKO from exceeding the standard within 60 days of simulated storage (6 days of oven storage). Notably, only ALG/GLN encapsulation could prevent the acid value of AKO from exceeding the standard during the 60-day simulated storage period.

Overall, these results demonstrate that the addition of GLN significantly extended the storage lifespan of ALG-encapsulated AKO and improved its storage quality. This could be attributed to two main factors: first, ALG/GLN microcapsules had a lower surface oil content compared to ALG-only microcapsules; moreover, ALG/GLN microcapsules encapsulated more astaxanthin, thereby enhancing the antioxidant capacity of the product [22].

## 4. Conclusions

The optimal encapsulation conditions were determined as follows: an ALG:GLN ratio of 2:1, a 9% CaCl_2_ coagulation bath, and a nozzle size of 750 μm with freeze dry. Under these conditions, the microcapsules achieved an oil-loading efficiency of 62.63% and a surface oil content of 19.21%. Incorporating GLN into ALG-based microcapsules can significantly enhance thermal stability and antioxidant capacity of AKO and extend its shelf-life.

Compared with papers about AKO encapsulation, for example, C.A. Ortiz Sánchezet al. encapsulated AKO using gum arabic, achieving an optimal encapsulation efficiency of 47.6715% with an astaxanthin content of 38.5726 μg/g [10]. also, our laboratory previously explored krill oil Pickering emulsions prepared by whey protein isolate and chitosan, which achieved an encapsulation efficiency of 80.43% and high ferric reducing antioxidant power (a 2.8-fold increase) [11]. the encapsulation efficiency in this study is at a moderate level [22].

Regarding oxidative stability, evaluated the levels of primary and secondary oxidation products in microcapsules of esterified AKO (EKO) during 25 days of storage at 25 °C. Their results showed that after encapsulation, the oxidation levels of EKO were slightly higher than those of non-encapsulated EKO, which might be attributed to the oxidation of surface oil caused by high temperature during spray drying [11]. In contrast, the microcapsules prepared in our study exhibited better oxidative stability, effectively inhibiting the increases in peroxide value and anisidine value during storage.

## Data Availability

The original contributions presented in this study are included in the article. Further inquiries can be directed to the corresponding author.
